# Prebiotics from Marine Macroalgae for Human and Animal Health Applications

**DOI:** 10.3390/md8072038

**Published:** 2010-07-01

**Authors:** Laurie O’Sullivan, Brian Murphy, Peter McLoughlin, Patrick Duggan, Peadar G. Lawlor, Helen Hughes, Gillian E. Gardiner

**Affiliations:** 1 Eco-Innovation Research Centre, Department of Chemical and Life Sciences, Waterford Institute of Technology, Waterford, Ireland; E-Mails: losullivan@wit.ie (L.O.S.); bmurphy@wit.ie (B.M.); pmcloughlin@wit.ie (P.M.); pduggan@wit.ie (P.D.); ggardiner@wit.ie (G.E.G.); 2 Teagasc, Pig Development Unit, Moorepark Research Centre, Fermoy, County Cork, Ireland; E-Mail: peadar.lawlor@teagasc.ie

**Keywords:** marine macroalgae, polysaccharides, prebiotics, human and animal health

## Abstract

The marine environment is an untapped source of bioactive compounds. Specifically, marine macroalgae (seaweeds) are rich in polysaccharides that could potentially be exploited as prebiotic functional ingredients for both human and animal health applications. Prebiotics are non-digestible, selectively fermented compounds that stimulate the growth and/or activity of beneficial gut microbiota which, in turn, confer health benefits on the host. This review will introduce the concept and potential applications of prebiotics, followed by an outline of the chemistry of seaweed polysaccharides. Their potential for use as prebiotics for both humans and animals will be highlighted by reviewing data from both *in vitro* and *in vivo* studies conducted to date.

## 1. Introduction

Marine macroalgae, or seaweeds as they are more commonly known, are one of nature’s most biologically active resources, as they possess a wealth of bioactive compounds. For example, compounds isolated from marine macroalgae have demonstrated various biological activities, such as antibacterial activity [1], antioxidant potential [2,3], anti-inflammatory properties [4], anti-coagulant activity [5], anti-viral activity [6] and apoptotic activity [7]. As a result, seaweed-derived compounds have important applications in a range of products in food, pharmaceuticals and cosmetics [8–10]. In addition to bioactive components, macroalgae are a rich source of dietary fiber (25–75% dry weight), of which water-soluble fiber constitutes approximately 50–85% [11]. Seaweeds are commonly classified into three main groups based on their pigmentation. Phaeophyta, or brown seaweeds, are predominantly brown due to the presence of the carotenoid fucoxanthin and the primary polysaccharides present include alginates, laminarins, fucans and cellulose [12,13]. Chlorophyta, or green seaweeds, are dominated by chlorophyll a and b, with ulvan being the major polysaccharide component [14]. The principal pigments found in rhodophyta, or red seaweeds, are phycoerythrin and phycocyanin and the primary polysaccharides are agars and carrageenans [15].

In the past decade, considerable research has been conducted on dietary modulation of intestinal microbiota. One particular area of research has focused on the concept of dietary “prebiotics” as functional ingredients for gut health, both for humans and animals. This review will examine evidence that polysaccharides from marine macroalgae, such as fucoidan, laminarin, alginate and their derivatives, may offer potential for use as prebiotics, with particular emphasis on their use in human and animal health applications. First the concept of prebiotics will be introduced, followed by a summary of the chemistry of seaweed polysaccharides, and a discussion of data from both *in vitro* and *in vivo* studies that have examined the prebiotic potential of seaweed polysaccharides.

## 2. The Prebiotic Concept

**Figure 1:**
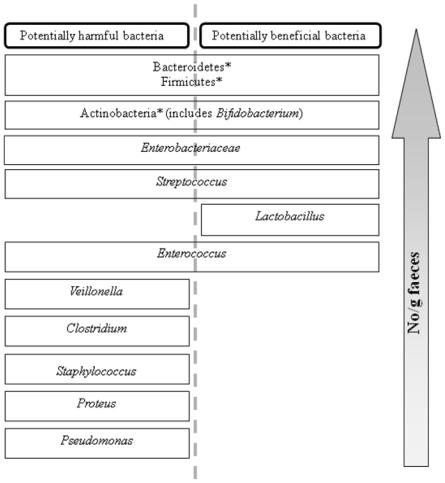
Distribution of the dominant, sub-dominant and minor components of human fecal microflora. Major dominant phyla are denoted. *: Other components are at the family or genus level (adapted from reference [16]).

The gastrointestinal tract (GIT) of both humans and animals is a complex, diverse, microbial ecosystem. The colon is the most heavily colonized region of the GIT with up to 10^12^ bacteria per gram of intestinal contents. The dominant genera include *Bacteroides*, *Prevotella*, *Eubacterium*, *Clostridium* and *Bifidobacterium*, with *Lactobacillus*, *Staphylococcus*, *Enterococcus*, *Streptococcus*, *Enterobacter* and *Escherichia* part of the sub-dominant flora (Figure 1) [16]. Potentially pathogenic and beneficial bacteria co-exist (Figure 1); however, strategies are being sought to influence this composition towards a more favorable balance, by reducing the amount of potentially harmful or pathogenic species and promoting the growth of species thought to have beneficial effects on host health [17]. Dietary modulation of the intestinal microflora can either be achieved via oral administration of probiotic micro-organisms or prebiotic compounds. The prebiotic concept was first proposed by Gibson and Roberfroid in 1995 [17] and the most recent definition of a prebiotic is “a selectively fermented ingredient that allows specific changes, both in the composition and/or activity of the gastrointestinal microflora that confers benefits upon the host wellbeing and health” [18]. To be considered prebiotic, a compound must satisfy a number of criteria; firstly it must be resistant to digestion in the upper GIT and therefore resistant to acid and enzymatic hydrolysis; secondly, it must be a selective substrate for the growth of beneficial bacteria and therefore result in a shift in the profile of the microflora and finally, it must induce luminal or systemic effects that are beneficial to host health [18]. In theory, any carbohydrate that enters the colon can potentially be considered prebiotic. However, while many naturally occurring carbohydrates from sources such as fruits and vegetables (*i.e.*, chicory, artichoke, garlic, bananas) and milk have been investigated both *in vitro* and *in vivo* [19]; evidence that the compound satisfies the prebiotic criteria outlined above must ultimately be obtained in well-controlled human/animal studies. To date, only three carbohydrates types are accepted as true prebiotics; inulin and oligofructose, galactooligosaccharides and lactulose [20]. Many polysaccharides from various sources have displayed prebiotic activity both *in vitro* and *in vivo*. As seaweeds are rich in polysaccharides, they are an obvious choice for investigation as a source of prebiotics.

### Applications of prebiotics

**Figure 2:**
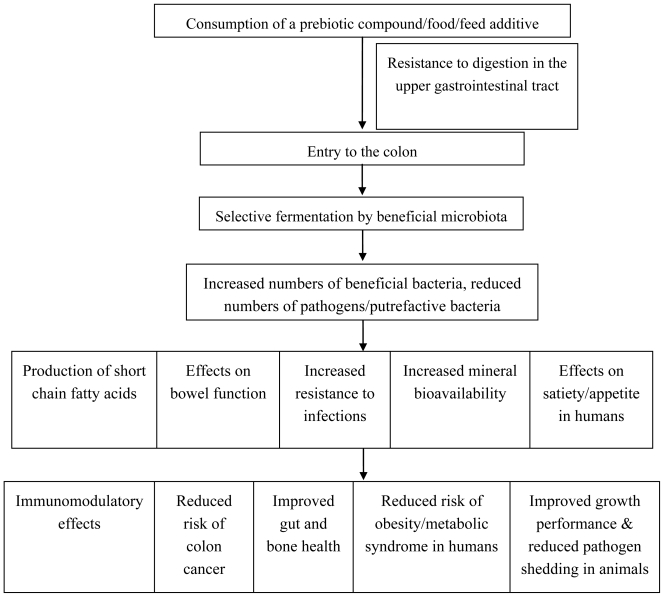
Mode of action of prebiotics and purported health benefits in humans and animals.

There are numerous purported health benefits attributed to the consumption of prebiotics by both humans and animals (Figure 2). The most notable and direct effects of prebiotics *in vivo* are mediated via modulation of intestinal microbiota (Figure 2) populations. A number of health effects in humans can be attributed to modulation of gut microflora and these have been extensively studied and reviewed [21–26]. Prebiotics can be consumed as dietary supplements or in functional foods. A functional food is defined as a food which provides a health benefit beyond basic nutrition [27]. At present, there are a number of functional foods on the market which contain prebiotic compounds; for example, infant formula, soy milk, breakfast cereals and yogurts [28].

Prebiotic compounds may also be added to animal feed, as an alternative to antibiotics. Sub-therapeutic doses of antibiotics were used in-feed in Europe and continue to be used in the US as a management tool to promote growth and maintain health in farm animals, in particular pigs and poultry. However, due to concerns over increasing bacterial antibiotic resistance, in-feed antibiotics are no longer permitted for use as growth promoters in the EU since 2006. Consequences, including increased enteric infections, reduced pig performance and increased mortality have been seen in Nordic countries where the antibiotic ban has been in place since the late 1990s [29]. Therefore, one of the challenges facing the livestock industry is maintaining the growth performance targets required for Consumption of a prebiotic compound/food/feed additive Resistance to digestion in the upper gastrointestinal tract Entry to the colon Selective fermentation by beneficial microbiota Increased numbers of beneficial bacteria, reduced numbers of pathogens/putrefactive bacteria Production of short chain fatty acids Effects on bowel function Increased resistance to infections Effects on satiety/appetite in humans Increased mineral bioavailability Immunomodulatory effects Reduced risk of colon cancer Improved gut and bone health Reduced risk of obesity/metabolic syndrome in humans Improved growth performance & reduced pathogen shedding in animals cost-effective animal production without the use of antibiotic growth promoters. Although the exact mode of action of antibiotic growth promoters is unknown, they most likely act via modulation of intestinal microbial populations, including reduction of pathogenic microorganisms. Prebiotic compounds therefore offer potential as an alternative to in-feed antibiotic growth promoters [30]. Apart from improving animal performance and health, prebiotics may also reduce carriage of enteric pathogens, thereby preventing transmission to humans (Figure 2) [30].

Dietary prebiotics sourced from seaweeds may provide a means to modulate the intestinal microbiota thereby improving the overall health of animals and humans. In particular, seaweeds contain a high concentration of polysaccharides of varying structure and functionality which could potentially be exploited as prebiotics.

## 3. Chemistry of Seaweed Polysaccharides

The chemical structures of seaweed polysaccharides have been described extensively [31–35]. Contained primarily in the cell walls, key functions of these relatively high molecular weight polysaccharides include, acting as a food reserve, provision of strength and flexibility to withstand wave action, maintenance of ionic equilibrium in the cell and prevention of cell desiccation. The composition varies according to season, age, species, and geographic location [36]. They are found with a wide variety of chemical structures, but some general characteristics have been identified [34]. They are rich in hydroxyl (-OH) groups, making them hydrophilic and often water soluble, and are known to establish intra-chain H-bond networks, making them stiff and rigid and suitable as thickeners. The regularity of their structures also promotes interaction with external ions and inter-chain H-bonding (e.g., gelation). Key polysaccharides found in chlorophyta, phaeophyta and rhodophyta are described in the following sections.

### 3.1. Polysaccharides from chlorophyta

#### Complex sulfated hetero-polysaccharides

**Figure 3:**
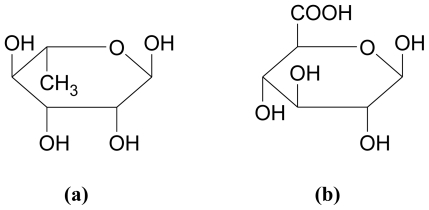
Green seaweed constituents: **(a)** α-L-rhamnose and **(b)** glucuronic acid [31].

The cell wall matrix of chlorophyta contains highly complex sulfated hetero-polysaccharides [37]. The extracted polysaccharides from *Ulva* spp. (12% of the algal dry weight) have been reported to contain 16% sulfate and 15–19% uronic acids [38,39]. Each molecule of these heteropolysaccharides is made up of several different residues, with the major sugars being glucuronic acid, rhamnose, arabinose and galactose, in a variety of combinations [31]. Figure 3 shows the structures of rhamnose and glucuronic acid, two of the constituent sugars of green seaweed polysaccharides.

The polysaccharide ulvan is easily extracted from *Ulva rigida* [40,41]. It is composed of β-(1,4)-xyloglucan, glucuronan and cellulose in a linear arrangement [31,41]. It corresponds to a water-soluble dietary fiber and is resistant to both human digestive tract enzymes and degradation by colonic bacteria. This polysaccharide cannot therefore be considered prebiotic; however, it could potentially be hydrolysed to bioactive oligosaccharides [42].

### 3.2. Polysaccharides from phaeophyta

#### 3.2.1. Alginates (also called alginic acid or algin)

Alginic acid is an anionic polysaccharide that occurs in all brown algae. It is the most abundant cell wall polysaccharide in brown algae [43]. For example, the alginate content of a number of seaweeds, based on dry weight, is as follows: *Ascophylum nodosum*, 22–30%; *Laminaria digitata* fronds, 25–44%; *L. digitata* stipes, 35–47%; *L. hyperborea* fronds, 17–33%; *L. hyperborea* stipes, 25–38% [34]. Alginate contents of between 17 and 45% have been reported in *Sargassum* spp. [44,45]. The industrial process for the extraction of alginates from brown seaweeds is as follows [35]:

- The seaweed is washed, macerated, extracted with sodium carbonate and filtered
- Sodium/calcium chloride is added to the filtrate and a fibrous precipitate of sodium/calcium alginate is formed
- The alginate salt is transformed to alginic acid by treatment with hydrochloric acid
- The alginate is purified, dried and powdered

**Figure 4:**
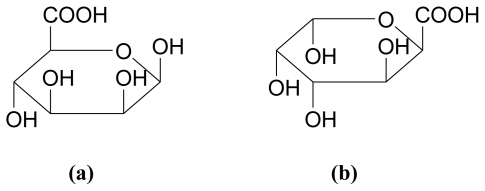
Constituent acids of alginic acid, where **(a)** is β-D-mannuronic acid and **(b)** is α-L-guluronic acid [43].

Alginic acid is a linear polysaccharide containing 1,4-linked β-D-mannuronic acid (M) and α-L-guluronic acid (G) residues, arranged in a non-regular block-wise order along the chain [46]. The residues typically occur as (-M-)_n_, (-G-)_n_ and (-MG-)_n_ sequences or blocks [43]. The carboxylic acid dissociation constants have been determined as pK_a_ = 3.38 and pK_a_ = 3.65 for M and G, respectively, with similar values obtained for the polymers [47]. Figure 4 illustrates the structure of β-D-mannuronic acid and α-L-guluronic acid.

Mannuronic and guluronic acids are classed as uronic acids. The uronic acids are simple monosaccharides in which the primary hydroxyl group at C_6_ has been oxidized to the corresponding carboxylic acid. Their names retain the root of the monosaccharides, but the *-ose* sugar suffix is changed to *-uronic acid*. For example, galacturonic acid has the same configuration as galactose, and the structure of glucuronic acid corresponds to that of glucose. Uronic acids are present in all three algal divisions [31].

#### 3.2.2. Fucans/Fucoidans

**Figure 5:**
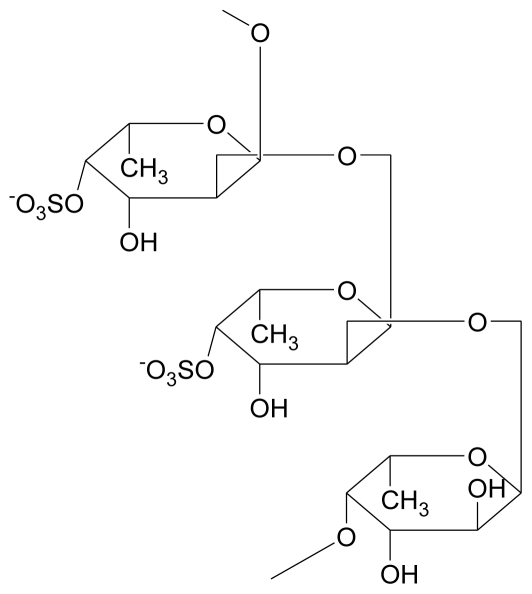
Fucoidan: Branched polysaccharide sulfate ester with L-fucose building blocks as the major component with predominantly α-(1,2) linkages [43].

Fucoidans are a complex series of sulfated polysaccharides found both intercellularly and in the cell wall of brown algae, with molecular weights typically of the magnitude 1,000,000. They play a role in cell organization and may be involved in the cross-linkage of alginate and cellulose [34]. Fucoidan is a water-soluble branched matrix polysaccharide sulfate ester, with L-fucose building blocks as the major component with predominantly α-(1,2) linkages [48]. Brown algae contain 5–20% fucoidan [44], about 40% of which is sulfate esters. The basic structure of fucoidan is shown in Figure 5.

Fucoidans are reported to display physiological and biological activities, including anticoagulant, antithrombotic, antiviral, antitumor, immunomodulatory, antioxidant, and anti-inflammatory [49], with therapeutic potential increasing with the degree of sulfation [34]. In addition, they protect gastric mucosa against the proteolytic activity of gastric juice. Furthermore, studies have demonstrated that fucoidan may prevent *Helicobacter pylori* infection in the stomach, thereby reducing the risk of associated gastric cancers [50].

#### 3.2.3. Laminarin

**Figure 6:**
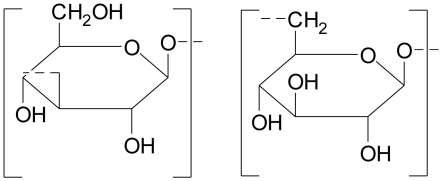
Basic chemical units of laminarin, made up of β-(1,3) and β-(1,6) linked glucose.

Laminarin is a relatively low molecular weight storage polysaccharide found commonly in brown algae. It may constitute up to 35% of the dried weight of the seaweed [34]. A member of the 1–3-β-D-glucan family, laminarin, consists of a linear structure with a small degree of side branching [51]. It is composed of (1,3)-β-D-glucopyranose residues, in which some 6-*O*-branching in the main chain and some β-(1,6) intrachain links are present (Figure 6 and Table 1) [52]. Laminarins from different algal species may vary in structural features; for example the degree of branching, the degree of polymerization (up to 50 carbohydrate residues, commonly about 25), and the ratio of (1,3)- and (1,6)-glycosidic bonds [52]. Most laminarins form complex structures that are stabilized by inter-chain hydrogen bonds and are therefore resistant to hydrolysis in the upper GIT and are considered as dietary fibers [53].

Laminarin has been identified as a modulator of intestinal metabolism through its effects on mucus composition, intestinal pH and short chain fatty acid (SCFA) production, especially butyrate [54,55] (see Section 4.3).

### 3.3. Polysaccharides from rhodophyta

Most red algal polysaccharides are galactans in which α-(1,3) and β-(1,4) links alternate [31]. Variety in the polysaccharides comes from sulfation, pyruvation and methylation of some of the hydroxyl groups and from the formation of an anhydride bridge between C_3_ and C_6_.

#### 3.3.1. Agar

**Figure 7:**
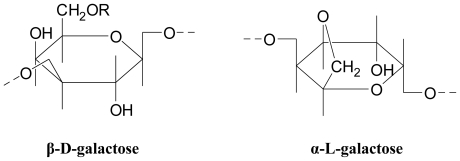
Agar constituents [31] (R=H or CH_3_).

Agars, the gel-forming polysaccharides extracted from certain families of rhodophyta, mainly Gracilariaceae and Gelidiaceae [56], are linear polymers with a sugar skeleton consisting of alternating 3-linked β-D-galactopyranosl and 4-linked 3,6-anhydro-α-L-galactopyranosyl units [57]. The basic structure of the constituent galactose residues is shown in Figure 7. Agar may be fractionated into two components [58]:

- Agarose (the gelling fraction)—A neutral linear molecule, free of sulfates
- Agaropectin (the non-gelling fraction)—Contains all the charged polysaccharide components, with some galactose residues substituted with pyruvic acid ketal, 4,6-*O*-(1-carboxyethylidene)D-galactopyranose, or methylated or sulfated sugar units [58]. Agaropectin is a slightly branched heterogeneous mixture of smaller molecules.

The ratio of the two fractions varies according to seaweed species and environmental conditions, and will affect the physiochemical, mechanical and rheological properties of agar. The molecular weight of agarose is about 120,000. Due to the substitutions in agaropectin, the molecular weight of agar is typically higher (up to 250,000), with a wide distribution [58].

Agarose is widely used as a gelling agent in microbiological media and for biotechnological applications [34]. Hu *et al.* [59] found that neoagaro-oligosaccharides (NAOS), obtained from the enzymatic hydrolysis of agarose, demonstrated prebiotic activity which was dependant on the degree of polymerization (see Section 4.2).

#### 3.3.2. Carrageenans (sulfated polysaccharides)

**Figure 8:**
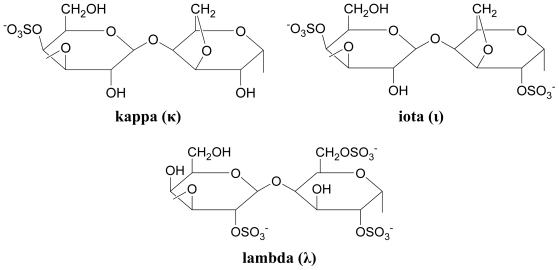
Basic structure of kappa-, iota-, and lambda-carrageenan [60].

In carrageenans, β-D-galactose alternates with α-D-galactose, not α-L-galactose as in agars. The degree of sulfation in carrageenans is generally much greater than that in agars. Based on physical properties, carrageenans are commonly classified into three types; kappa (strong rigid gels), iota (soft elastic gels) and lambda (thickening polymer) [34]. The basic structures of the three classes are shown in Figure 8.

Carrageenans are used in a wide variety of applications; for example, as thickening, stabilizing and encapsulation agents. It has been reported that degraded carrageenans may cause ulcerations in the GIT and gastrointestinal cancer [34].

#### 3.3.3. Other polysaccharides and polysaccharide derivatives

The presence of uronic acids in red seaweed polysaccharides has been reported. For example, a neutral xylan and a xylogalactan, with 4.8% uronic acids, were obtained from *Palmaria decipiens* [32]. Errea *et al.* [61] reported unusual structures in polysaccharides from the red seaweed *Pterocladiella capillacea*. Structural analysis indicated the presence of xylogalactans, with a low content of 3,6-anhydrogalactose and low molecular weight. The polysaccharides varied in the degree of xylopranosyl and sulfate substitution. The presence of 3-substituted, 4-linked D-galactopyranosyl residues was also reported.

A number of potentially useful sulfated polysaccharides from lesser-known red seaweed species have been reported [34]:

- Hypneans are extracted from the *Hypnea* spp. Structurally, hypneans are similar to agar and carrageenan, but with a higher percentage of 3,6-anhydrogalactose. They are primarily used as gelling agents in food applications and as fertilizers in dry arid soils.
- Porphyran is a highly substituted polysaccharide extracted from the *Porphyra* genus. It is used as a gelling agent, a nutritional supplement (e.g., to help cope with stress) and an antioxidant.
- Funorans, extracted from species such as *Gloiopeltis complanata*, are composed of a heterogeneous series of polysaccharides and sulfated galactans. Funoran has been shown to inhibit the adherence and colonization of oral bacteria, reducing dental caries in rat studies [62]. It is also reported to reduce blood pressure, lower cholesterol and exhibit anti-tumor properties.

Oligosaccharides are commonly defined as carbohydrate molecules with a low degree of polymerization (between 2 and 25) [19]. These molecules may be found naturally or derived from larger polysaccharides. Examples of depolymerization methods for algal polysaccharides include; free radical depolymerization for fucoidan, thermal degradation and enzymatic hydrolysis for alginate and chemical degradation for ulvan. While numerous studies have reported prebiotic activity for plant-derived oligosaccharides [19], few studies have specifically examined the effects of algal-derived oligosaccharides (see Section 5.1).

## 4. *In Vitro* Studies Examining the Prebiotic Potential of Seaweed Polysaccharides and Oligosaccharide Derivatives

While prebiotic activity must ultimately be determined *in vivo*, *in vitro* studies are useful for preliminary screening of candidate compounds and can generate information on functional mechanisms. The three main criteria that are tested *in vitro* are; non-digestibility, fermentability and selectivity [20]. Studies that have evaluated seaweeds and/or seaweed polysaccharides *in vitro* will be reviewed here.

### 4.1. Resistance to digestive enzymes

As discussed in Section 2, a prebiotic compound must be resistant to digestion in the upper GIT so that it can reach the lower intestine intact [18]. This can be investigated *in vitro* by testing resistance to acidic and enzymatic hydrolysis. Hu *et al.* [59] showed that agarose-derived NAOS were resistant to amylolytic enzymes by demonstrating via electrophoretic analysis that the compounds remained intact after 24 h incubation with an enzyme mixture. Similarly, glycerol galactoside, determined to be the fermentable component of the red alga *Porphyra yezoensis*, was not digested by salivary, gastric, pancreatic or intestinal enzymes [63]. Neither was it absorbed across excised segments of rat small intestine. A study by Deville *et al.* [55] showed that laminarin remained intact following incubation *in vitro* with HCl, human saliva and human gastric, pancreatic, small intestinal and colonic homogenates.

### 4.2. Selective fermentation by pure cultures

One of the criteria that a compound must meet in order to be classified as a prebiotic is “fermentation by intestinal microbiota” [18]. Compounds should be selectively fermented, *i.e.*, fermentable by beneficial species but not pathogens (Figure 1). This can be determined *in vitro* by incubating pure cultures of representative beneficial bacteria (usually *Lactobacillus* and *Bifidobacterium* spp.) as well as potentially pathogenic species (e.g., *E. coli*, *Enterococcus*) with the compound and investigating bacterial growth [18]. The usual procedure is to inoculate a range of bacterial strains into a microbiological medium in which the carbon source has been replaced by the test compound and measure growth in comparison to a glucose control. While many studies have evaluated potential prebiotics in this way [16], few have evaluated seaweed-derived compounds. However, studies conducted more than three decades ago showed that laminarin and alginate could be degraded by human colonic bacteria [64,65]. Hu *et al.* [59] showed that NAOS obtained from agarose were fermented by *Lactobacillus* and *Bifidobacterium* but not *E. coli* or *Enterococcus*. Increased growth rates, as determined by plate counts and pH decrease, were observed in comparison to fructo-oligosaccharides (FOS) but glucose was not included as a control. Nori, a dried form of the red seaweed *Porphyra yezoensis*, was fermented by all but one of five intestinal *Bifidobacterium* strains, but only when it had a high protein content [63]. In a subsequent experiment with 17 bacterial strains, glycerol galactoside, which was determined to be the fermentable component, was comparable with glucose as a substrate for *Bifidobacterium*. It was also fermented by *Bacteroides*, *Clostridium* and *E. coli*, albeit not as well as glucose but failed to stimulate the growth of *Enterococcus*, *Eubacterium* or *Lactobacillus* spp.

These results, while useful, should be interpreted with caution, as the test strains and conditions used may not be representative of those found within the intestinal tract. Therefore, these types of assays are recommended only as a screening tool in preliminary investigations [18].

### 4.3. Fermenter studies to determine effects on intestinal microbiota

A more meaningful way to assay potential prebiotic compounds is to test them in anaerobic fermenter systems inoculated with fecal/intestinal material [66]. This not only tests the ability of the intestinal microflora to ferment the compound but also evaluates stimulatory effects on microbial growth and/or activity. Effects on microbial growth can be monitored by culturing fermenter samples on selective media; however, culture-independent approaches employing molecular methods such as fluorescece *in situ* hybridization (FISH), polymerase chain reaction (PCR), denaturing gradient gel electrophoresis (DGGE) and 16S rRNA gene sequencing provide a more complete representation of microbial diversity [18]. Microbial activity is usually evaluated by measuring metabolic end products, such as SCFA and gases.

Total algal fibers extracted from whole *Himanthalia elongata*, *L. digitata* and *Undaria pinnatifida* were fermented by human fecal microflora after 24 h in a batch system, as determined by substrate disappearance, but were not completely metabolized to SCFA compared with sugarbeet fiber [67]. In a subsequent experiment, purified laminarins were highly fermented after an initial lag period (explained by the time required for induction of bacterial enzymes), alginates were fermented but not completely degraded to SCFA and fucans were not fermented at all. However, effects on microbial counts were not evaluated, so no conclusions on prebiotic activity can be made.

Deville *et al.* [54] showed that laminarin (either extracted from *L. saccharina* or *L. digitata*) was almost completely fermented in a human fecal batch fermenter system after an initial lag period, as measured by its disappearance over 48 h. Turbidity increases and pH decreases (both indicative of bacterial growth) were comparable in vessels containing laminarin, FOS or glucose after 24–48 h. Total SCFA concentrations were higher for *L. digitata* laminarin in comparison to glucose with higher amounts of propionate and butyrate observed. SCFA were, however, not measured for the *L. saccharina-*derived laminarin or FOS treatments. Furthermore, while FOS increased culturable *Lactobacillus* and decreased *Bacteroides*, laminarin had no effects on bacterial counts. Although PCR was used to confirm counts, no culture-independent analyses were performed. Similarly, SCFA concentrations increased in a human fecal batch culture on addition of laminarin or alginate compared to a control [68]. Reduced concentrations of potentially harmful microbial end products (ammonia, indole compounds, phenol compounds) were also observed. Taken together, this data was interpreted as an indication of fermentation of the seaweed polysaccharides by intestinal bacteria but, as is the case in many studies, no microbiological analyses were performed. However, Dierick *et al.* [69] demonstrated that dried whole *Ascophyllum nodosum* decreased potential pathogens (*E. coli*, streptococci) and total anaerobes in batch systems inoculated with either porcine small intestinal or cecal suspensions. This inhibitory activity may have been due to the reduction in pH, as the pH was not controlled during fermentation. However, a decrease in beneficial bacteria (*i.e.*, *Lactobacillus*) was also observed. The authors concluded that this intact brown seaweed is not a suitable fermentation substrate, however, further experiments were not conducted to evaluate this and only culturable microflora was investigated. Furthermore, the control vessel contained a synthetic diet which would have supplied all of the nutrients necessary for microbial growth, while the experimental vessels contained seaweed only. In practice, diets would be supplemented with *A. nodosum*, rather than consisting entirely of seaweed.

Michel *et al.* [70] evaluated alginate- and laminarin-derived oligosaccharides in continuous as well as batch human fecal fermentations compared with FOS. While total concentrations of SFCA did not differ between treatments, propionate production was increased by all of the test compounds relative to FOS. These and additional data reported in the study indicated that the oligosaccharides tested were fermented by the fecal microflora [70]. However, oligosaccharides from either source did not alter counts of *Bacteroides*, *Lactobacillus*, total anaerobes or aerobes and, in fact, *Bifidobacterium* counts were reduced ~1000-fold. The latter is in keeping with the reductions in *Lactobacillus* observed by Dierick *et al.* [69].

The most promising evidence of prebiotic activity of algal polysaccharides comes from a recent study that evaluated the effects of a dietary supplement containing a mixture of plant polysaccharides including *Undaria pinnatifida* fucoidans on human fecal microflora in a three-vessel colon model [71]. No control vessels were used; instead there was a control period before supplement addition and a washout period after. The product tested was predominantly fermented in the distal colon vessel. Although no effects on total SCFA were observed before, during or after supplement addition, lactate and butyrate were decreased in the ascending and descending colon vessels, respectively during the treatment period. Although some increases in individual SCFA were observed during the washout period (most notably butyrate in the distal colon), a concomitant increase in ammonium, a potentially toxic metabolite, was observed in the descending colon during treatment. It is encouraging to note that increases in *Bifidobacterium* were seen, both via plate counts and quantitative PCR (qPCR). Furthermore, DGGE revealed compositional changes within bifidobacterial populations. Cultivable *Lactobacillus* were increased but qPCR failed to show such an effect, although it did reveal increases in the *Bacteroides-Prevotella* group. Taken together, these data demonstrate that the product tested appears promising as a prebiotic; however, as it was a mixture of plant-derived polysaccharides, no conclusions can be made regarding prebiotic activity of the seaweed fucoidan component.

Overall, a number of studies mainly conducted in batch fermentation systems have demonstrated that seaweed polysaccharides are fermented by the intestinal microflora but few demonstrate selective stimulation of beneficial intestinal microbial populations. Additional studies in multi-chamber continuous culture systems are needed, as these replicate different gastrointestinal regions more closely. Even these systems suffer limitations, as an increase in a limited number of bacterial genera within a complex mixture is not definitive proof of a prebiotic effect. Further studies utilizing detailed molecular methods, such as metagenomics, are required in order to fully elucidate the impact of algal polysaccharides on the entire intestinal microbiome.

## 5. *In Vivo* Studies Examining the Prebiotic Potential of Macroalgal Polysaccharides and Oligosaccharide Derivatives

*In vitro* studies give an indication of the prebiotic potential of algal polysaccharides. However, *in vivo* studies are necessary to demonstrate prebiotic activity before worthwhile conclusions may be drawn. While there is substantial evidence in the literature to suggest that certain polysaccharides such as inulin and FOS have modulatory effects on the gut microflora in humans, to date no human trials have been conducted on polysaccharides from seaweeds. Studies have, however, been conducted in laboratory animals to determine prebiotic properties of seaweed polysaccharides with a view to establishing suitability for applications in other animals and humans. Feeding trials have also been performed in farm animals to investigate effects on animal health and growth performance.

### 5.1. Studies in laboratory animals

Wang *et al.* [72] demonstrated that rats fed test diets supplemented with 2.5% alginate oligosaccharides displayed a selective increase in the numbers of *Bifidobacterium* and *Lactobacillus* in both the cecum and feces. The prebiotic effect was greater than that observed in the control group which was fed a diet containing FOS, a well-established prebiotic. A study conducted by Hu *et al.* [59] demonstrated similar increases in fecal *Lactobacillus* and *Bifidobacterium* populations after feeding mice diets supplemented with either 2.5% or 5% agarose hydrolysate (NAOS) compared with a control diet or a diet containing FOS. The authors also demonstrated that the NAOS-fed group had increased *Lactobacillus* counts in the cecum seven days post-administration compared to the control group; however, cecal *Lactobacillus* counts in the test group were similar to those in the group fed FOS. In addition, the NAOS-fed group had lower numbers of *Bacteroides* compared to the control group. Overall, the NAOS-fed groups demonstrated large increases in populations of beneficial bacteria, with no adverse effects on animal health, suggesting that NAOS could be a potential algal prebiotic. Kuda *et al.* [68] reported that dietary supplementation with 1% laminarin resulted in an increase in *Bifidobacterium* counts in the cecum of rats compared to a control diet, but there was no significant difference in *Lactobacillus* counts. In the same study, laminarin was shown to suppress indole, cresol and sulfide, which are putrefactive compounds considered risk markers for colon cancer. Further evidence that laminarin is fermented by the intestinal microbiota was found in a study which showed that laminarin was not detected in the feces of rats fed laminarin [55]. A study by Gudiel-Urbano and Goni [73] demonstrated that rats fed *Undaria pinnatifida* and *Porphyra ternera* extracts had lower bacterial enzyme activity in the cecum. This provides additional indirect evidence of the effects of seaweed extracts on the intestinal microflora. Furthermore, the enzymatic activities that were reduced are implicated in the conversion of procarcinogens to carcinogens, suggesting a possible link between seaweed extract intake and reduced risk of colon cancer (also implied from data generated by Kuda *et al.* [68], as outlined above). Neyrinck *et al.* [53] demonstrated that dietary supplementation with laminarin also has systemic effects, as it protected against lipopolysaccharide-induced liver toxicity in a rodent model of systemic inflammation. Dietary laminarin had immunomodulatory effects, which the authors suggest are due either to a direct effect of laminarin on immune cells or to an indirect effect via modulation of the intestinal microbiota. However, intestinal microbial populations were not measured so no conclusions can be made regarding the latter. Overall, from the studies conducted to date, it is evident that seaweed polysaccharides and oligosaccharide derivatives demonstrate prebiotic effects in rodents.

### 5.2. Studies in farm animals

Prebiotics have a role to play in animal health; they are assumed to stimulate the growth of beneficial bacteria, thereby improving intestinal health and stimulating growth performance, particularly following weaning. The post-weaning period in pigs is stressful due to factors such as separation from the sow and littermates, digestive disruption as a result of the sudden shift from milk to a cereal-based diet and exposure to a new environment [74]. These stressors can cause a microbial imbalance in the gut, leading to undesirable effects such as reduced feed intake, a consequential reduction in growth rate and increased susceptibility to post-weaning diarrhea and pathogenic infections [75]. Modulation of the gut microbiota by prebiotics may prove to be a useful strategy in promoting growth and may alleviate some of the undesirable effects observed in weanling pigs. Many studies have evaluated the effects of seaweed extracts and seaweed polysaccharides, such as laminarin in feeding experiments, mainly in pigs but also in lambs and cattle. Such experiments have been conducted as seaweed extracts and seaweed polysaccharides are considered good prebiotic candidates as they are resistant to digestion in the small intestine and are fermented by colonic microbiota, as outlined in section 4 [54,55]. However, the differences in digestive physiology and anatomy must be borne in mind when attempting to extrapolate data from ruminants (cattle and sheep) to pigs, which are monogastric. Effects on parameters, such as growth performance, nutrient digestibility, volatile fatty acids and intestinal microbiota have been studied in detail, particularly in relation to swine health and nutrition and will be reviewed below. Only statistically significant results (cut-off of P < 0.05) are discussed.

#### 5.2.1. Effects of marine polysaccharides on growth performance

Reilly *et al.* [76] found that dietary inclusion of seaweed extracts containing both laminarin and fucoidan had no effect on feed intake, weight gain, feed conversion ratio or nutrient digestibility in weanling pigs (Table 2). A lack of improvement in weight gain, feed intake and feed efficiency has also been reported in finishing lambs fed diets supplemented with Tasco 14^™^ (sun-dried whole *A. nodosum*) [77]. Furthermore, Gardiner *et al.* [78] found no effects on feed intake or feed conversion ratio but reported a decrease in weight gain when an *A. nodosum* extract was fed to grower-finisher pigs (Table 2). The authors suggested that the presence of anti-nutritional phyto-chemicals and a high level of chelated metals in the crude seaweed extract may have been responsible for the decreased growth performance. As a consequence, Gahan *et al.* [79] supplemented diets with a more refined seaweed extract containing only laminarin and fucoidan with a view to replacing lactose in the diet of weaned pigs. The authors extract increased feed intake and weight gain and improved feed conversion ratio in a manner similar to lactose (Table 2).

High health status of animals is often cited as a reason for lack of effects on growth performance. For this reason, McDonnell *et al.* [80] fed pigs a nutritionally challenged diet supplemented with laminarin and found increased weight gain and increased gain to feed ratio (equivalent to reduced feed conversion ratio). However, fucoidan had no effect on pig growth or daily feed intake. A more recent study conducted by O’Doherty *et al.* [81] reported that dietary inclusion of a laminarin-fucoidan extract increased average daily weight and gain to feed ratio in pigs fed diets formulated to create a nutritional challenge, similar to those fed by McDonnell *et al.* [80] (Table 2). Similarly, Turner *et al.* [82] observed a positive linear effect on feed intake and a quadratic effect on weight in young pigs challenged with *Salmonella* and fed an *A. nodosum* extract.

Overall, the evidence examining the effect of seaweed extracts on growth performance in farm animals is equivocal, a trend which is also observed in studies examining the effects of established prebiotics in animals [30]. Reasons for the differences in responses observed between studies may be due to factors such as, differences in dietary inclusion levels, variations in the types and purity of seaweed extracts evaluated and differences in the species, age and health status of animals used (Table 2). Additional studies examining the effects of purified polysaccharides from seaweeds are needed.

#### 5.2.2. Effects on intestinal microflora

In addition to the studies conducted in rodents (see Section 5.1), a number of research groups have examined the effect of feeding seaweed extracts on the gut microflora of farm animals. Dierick *et al.* [69] reported that inclusion of *A. nodosum* meal in the diets of weanling pigs resulted in lower numbers of *E. coli* in the small intestine. Furthermore, the authors reported that the small intestinal *Lactobacillus*/*E. coli* ratio was increased in the test group indicating a potentially beneficial shift in the microbial ecosystem. However, Gardiner *et al.* [78] reported that dietary supplementation with a crude *A. nodosum* extract in finisher pigs resulted in reduced ileal coliform counts but also reduced cecal bifidobacteria, which are considered beneficial (Table 2). Similarly Reilly *et al.* [76] reported reductions in enterobacteria but also bifidobacteria and lactobacilli in the cecum and/or colon of weaned pigs fed *L. digitata* containing laminarin and fucoidan. Similar effects were seen in a group fed *L. hyperborea* (also containing laminarin and fucoidan), but there were no reductions in enterobacteria. However, a combination of both extracts resulted in a reduction of enterobacteria and lactobacilli in the cecum and colon but no effects on bifidobacteria were observed (Table 2). Lynch *et al.* [83] found that finisher pigs offered diets supplemented with increasing amounts of an intact *L. hyperborea* extract had reduced cecal *Bifidobacterium* and colonic *Lactobacillus* counts [76]. In a subsequent experiment also in finisher pigs, dietary supplementation with a purer fucoidan extract increased *Lactobacillus* counts in the proximal and distal colon (Table 2). On the other hand, McDonnell *et al.* [70] demonstrated that weanling pigs offered diets supplemented with laminarin alone or laminarin in combination with fucoidan had reduced fecal *E. coli* counts compared to pigs fed diets without laminarin. However, laminarin alone or in combination with fucoidan did not affect fecal lactobacilli but fucoidan alone resulted in higher counts of fecal lactobacilli compared to control animals (Table 2). The authors suggest that fucoidan has antimicrobial activity, while laminarin exerts prebiotic activity. In another study, O’Doherty *et al.* [81] hypothesized that a laminarin-fucoidan seaweed extract might prevent any negative effects of lactose removal from weanling pig diets. Inclusion of seaweed extract in either high or low lactose diets resulted in decreased fecal *E. coli* counts compared to non-seaweed diets. Decreases were similar to those observed in pigs fed high lactose diets alone. Increased lactobacilli numbers were also observed in pigs fed diets supplemented with seaweed extract but only in combination with high lactose levels. Janczyk *et al.* [84] reported that dietary supplementation with alginate resulted in higher enterococci counts in the distal small intestine, cecum and proximal colon of weanling pigs after an 11-day administration period compared with inulin or a control diet. However, reductions in lactobacilli were also observed in all intestinal segments but only after 6 days of alginate supplementation, and not prior to or after this timepoint. Molecular analysis of the intestinal microbiota by DGGE also demonstrated higher microbial diversity in the distal small intestine of alginate-supplemented pigs compared to animals fed a control diet. To our knowledge this is the only study to date that has examined effects of a seaweed polysaccharide on the intestinal microflora at a molecular level. Dietary supplementation with prebiotic compounds is also promising as a means of reducing pathogen shedding in farm animals as a strategy to improve food safety. Braden *et al.* [85] examined the effects of dietary supplementation with 2% dried *A. nodosum* (Tasco 14^™^), on fecal enterohemorrhagic *E. coli* O157:H7 populations on cattle hides and in feces. The authors reported reductions in the prevalence of fecal *E. coli* O157 and O157:H7 in hide swabs and fecal samples of *A. nodosum*-supplemented animals compared to animals offered a control diet. Bach *et al.* [77] also examined the effect of feeding Tasco 14^™^ on *E. coli* O157:H7 shedding but in cattle deliberately challenged with the pathogen. *E. coli* O157:H7 detection was less frequent in fecal samples of animals fed diets supplemented with either 10 g/kg of Tasco 14^™^ for 14 days or 20 g/kg for seven days compared to animals fed the control diets or diets supplemented with 20 g/kg for 14 days. The exact mode of action of the seaweed extract was not identified in either study but inhibitory effects against *E. coli* were attributed to direct antimicrobial activity rather than a prebiotic effect. This was concluded due to a lack of effects on microbial metabolites in the feces, indicating that the seaweed extract did not alter intestinal microbial populations and was therefore not fermented; however, only fecal metabolites were measured and further studies are warranted to explore effects on colonic microflora.

Overall, while increases in beneficial intestinal microbial populations are seen in some experiments where animals were fed seaweed-extracts, there is evidence to show that feeding purer extracts containing known amounts of characterized polysaccharides may be more effective in achieving a prebiotic effect. On the other hand, some extracts result in decreases in Gram negative intestinal populations but are not selective, as concomitant decreases in populations considered beneficial are sometimes also observed. This is most likely due to a direct antimicrobial effect rather than prebiotic activity. It has been suggested that certain seaweed polysaccharides may exert inhibitory effects while others might stimulate microbial populations and for this reason polysaccharides should perhaps be fed in combination.

In addition to examining the effects of potential prebiotic compounds on gut microbial ecology, microbial activity can be elucidated by examination of fermentation end products such as SCFA. These are predominantly produced in the hindgut which is where they exert the majority of their effects. For example, within the colon, SCFA are a source of energy for the host but also have important effects on host epithelial physiology [86]. SCFA also stimulate gut integrity and lower intestinal pH, which is associated with a reduction in pathogen growth [87]. Lynch *et al.* [83] observed that a crude *L. hyperborea* extract resulted in changes in the concentration of total fatty acids and molar proportions of individual fatty acids in the cecum but not the proximal colon of finisher pigs. However, Reilly *et al.* [76] demonstrated that supplementation with a crude *L. hyperborea* extract increased the concentration of colonic volatile fatty acids, whilst an extract derived from *L. digitata* increased volatile fatty acids in the cecum of weanling pigs. Therefore, extracts from different seaweeds, although similar in laminarin and fucoidan content, are fermented in different areas of the GIT and this may be due to differences in the purity of these extracts. It is also apparent that results can also vary between studies even when the same seaweed is fed; this was perhaps due to the differing age of the animals used. In a subsequent experiment, Lynch *et al.* [83] examined the effects of purified laminarin, fucoidan and a combination of both on the concentration of fatty acids in the proximal and distal colon (but not the cecum) of finishing pigs. Interestingly, the fucoidan-supplemented diet increased total fatty acid concentrations in the proximal and distal colon; there were however, no changes in fatty acid concentrations in the proximal colon of pigs fed the crude seaweed extract, as outlined above. This indicates that the purified polysaccharides were broken down to a greater extent than the crude extract. McDonnell *et al.* [80] reported increases in fecal lactobacilli populations in weanling animals offered a laminarin-fucoidan extract, however, no changes in fecal fatty acids in this group were observed. Similarly, O’Doherty *et al.* [81] reported increases in *Lactobacillus* spp. in weanling pigs fed a laminarin-fucoidan extract in combination with a high lactose diet but reported no effects on total fecal volatile fatty acid concentrations or the fatty acid profile. However, measurement of fecal volatile fatty acids in pigs may not be indicative of the degree of fermentation in the large intestine, as the fatty acids produced in the cecum and colon will most likely have been absorbed there and hence will not be excreted in the feces.

#### 5.2.3. Additional benefits of prebiotics in swine husbandry

Dietary prebiotic supplementation in pigs may reduce nitrogen excretion, which may have positive environmental implications. Prebiotics, such as inulin have yielded a reduction in fecal ammonia concentrations in pigs [86]. While limited studies have examined this effect using polysaccharides from algal sources, Reilly *et al.* [76] reported a significant reduction in ammonia concentrations in the colon of pigs offered diets containing either a *L. hyperborea* extract or an *L. digitata* extract.

Boar taint is an undesirable taste or odor which can be evident when cooking or eating pork from non-castrated male pigs once they reach puberty. It is mainly caused by an accumulation of skatole and androstenone in adipose tissues. Skatole is produced by bacterial fermentation of tryptophan in the hindgut of animals [87]. Where slaughter weight is high and consequentially pigs are older at slaughter, as is the case in North America and continental Europe, boar taint is usually controlled by castration. Countries like Ireland and the UK still produce entire male pigs since slaughter weight/age has traditionally been low in these countries. However, as slaughter weight and hence age at slaughter is increasing in Ireland and the UK and there is European pressure to ban the practice of castration, alternative nutritional means of mitigating against boar taint are being investigated. Lanthier *et al.* [88] demonstrated that animals offered a diet containing inulin had significantly reduced plasma, cecal and fat skatole concentrations compared to animals offered a control diet. The authors suggest that this is possibly due to a reduction in hindgut proteolytic bacteria in favor of carbohydrate-fermenting bacteria. In addition, Byrne *et al.* [89] demonstrated that feeding pigs chicory root, a rich source of inulin, resulted in a significant reduction in boar taint sensory characteristics and reduced blood and back fat concentrations to below detectable levels. To the best of our knowledge, no studies have been conducted to date to assess the effects of marine polysaccharides on skatole or adrostenone levels in pigs.

## 6. Conclusions

Seaweed grows in abundance in coastal areas and may be a source of compounds which could be exploited for novel functional ingredients for human and animal health applications. This review has examined the evidence from both *in vitro* and *in vivo* experiments and found that seaweed-derived polysaccharides may have prebiotic activity. While a substantial amount of research has been conducted to date on animals, no studies have been conducted in human subjects to date. Results from *in vivo* studies using laboratory animals and domestic pigs are promising. However, it is difficult to reach consensus on the benefits because of the conflicting results obtained in different studies. This is most likely due to a number of factors such as, experimental conditions, intra-laboratory variations in age and physiology of the animals studied, variations in the type, purity and dose of seaweed extracts fed, differences in the concentration of active compounds within seaweed extracts and differences in dietary inclusion of other feed ingredients. In addition to these factors, seasonal variations in the composition of seaweed polysaccharides also require consideration when feeding intact seaweed meal. Future work in the area of seaweed-derived prebiotics should aim to examine the effects of purified seaweed polysaccharides on gut morphology and intestinal microbiota in parallel with well-established prebiotics, such as FOS or inulin. Furthermore, the role of seaweed prebiotics in animal husbandry may go beyond that of health applications with potential for improvement of environmental pollution and meat quality.

## Figures and Tables

**Table 1:** Chemical structure of laminarins from various seaweeds [34].

Seaweed	Structure of Laminarin
Several species of *Laminaria*	Linear β-(1,3) linked D-glucose
*Laminaria digitata*	Linear backbone of β-(1,3) linked D-glucose, with β-(1,6) linked side chains
*Eisenia bicyclis*	Linear chain of (1–3) and (1–6) links, in the ratio of 2:1

**Table 2:** Effects of algal prebiotics on pig health.

Algal supplement	Age and heath status of pigs	Dose	Effect on gut microbiota	Effect on growth performance and health	Ref
*A. nodosum* extract (ANE)	Healthy grower-finisher pigs	0, 3, 6 or 9 g/kg basal feed	Animals supplemented with 6 or 9 g ANE/kg had lower (P < 0.05) ileal coliform counts than animals that received 3 g/kg Linear reduction (P < 0.05) in coliform counts in the ileal contents as ANE increased Reduction (P < 0.05) in cecal *Bifidobacterium* counts with increasing ANE supplementation	Linear decrease (P < 0.05) in weight gain with increasing levels of extract	[78]
*A. nodosum* meal (ANM)	Healthy, weanling piglets	10 or 20 g/kg basal feed	Reduced (P < 0.05)*E. coli* in the small intestine and increased (P< 0.05) *Lactobacillus*/*E. coli* ratio in animals fed 10 g/kg	No effects on final weight No effects on intestinal histology or intestinal immune cells	[69]
*Laminaria* spp. extract containing a combination of laminarin & fucoidan (ranging from 0.112–0.446 and 0.890–0.356 g/kg, respectively)	Healthy, weanling piglets	0, 1, 2, 4 g/kg basal feed containing increasing levels of lactose (60–250 g/kg)	Effects on gut microbiota were not determined	Weight gain and feed intake increased (P < 0.05) as the level of seaweed extract increased; however, this was only observed when fed in combination with low and medium levels of lactose	[79]
*Laminaria* spp. extract containing either laminarin or fucoidan or a combination of both (0.3 and 0.24 g/kg, respectively)	Healthy, weanling piglets fed a nutritionally-challenged diet (high protein, low lactose)	Basal feed + 0.3 g/kg laminarin; basal feed + 0.24 g/kg fucoidan; basal feed + 0.3 g/kg laminarin and 0.24 g/kg fucoidan	Laminarin supplementation resulted in lower (P < 0.05) fecal *E. coli* populations compared to control group Interaction (P < 0.01) between laminarin and fucoidan with respect to fecal lactobacilli populations	Laminarin supplementation resulted in increased (P < 0.01) daily weight gain Pigs offered combination of laminarin and fucoidan had reduced (P < 0.05) diarrhoea	[80]
Alginate	Healthy, weanling piglets	1 g/kg starter feed	Higher enterococci counts in distal small intestine, cecum and proximal colon (P < 0.001) compared with inulin or control group. Reduced (P < 0.05) lactobacilli in all intestinal segments but only after 6 days of alginate supplementation, and not before or thereafter Increased microbial diversity	Animals were in good health throughout the study	[84]
Exp 1: *L. hyperborea* extract (112 g/kg laminarin & 89 g/kg fucoidan) Exp 2: Purified laminarin (0.30 g/kg), fucoidan (0.24 g/kg) and a combination of both laminarin and fucoidan (0.30 and 0.24 g/kg, respectively)	Healthy finishing boars	Exp 1:0.7, 1.4, 2.8, 5.6 g/kg extract Exp 2: Basal diet + 0.30 g/kg laminarin; basal diet + 0.24 g/kg fucoidan; basal diet + 0.30 g/kg laminarin and 0.24 g/kg fucoidan	Exp 1: Quadratic response (P < 0.05) to seaweed extract on cecal (P < 0.05) *Enterobacterium* spp., colonic (P < 0.05) *Enterobacterium* spp. and (P < 0.001) *Bifidobacterium* spp. Linear decrease in cecal *Bifidobacterium* spp and colonic *Lactobacillus* spp. with increasing seaweed extract (P < 0.01, P < 0.05, respectively) Exp 2: Fucoidan diet resulted in increases in colonic *Lactobacillus* spp. (P < 0.001). Combination diet resulted in increase in *Enterobacterium* spp. (P < 0.05).	Growth performance was not evaluated	[83]
*L. hyperborea* extract (LHE), containing laminarin and fucoidan (0.17 and 0.13 g/kg, respectively) *L. digitata* extract (LDE), containing laminarin and fucoidan (0.17 and 0.14 g/kg, respectively) Combination of LHE and LDE containing laminarin and fucoidan (0.17 and 0.13 g/kg, respectively)	Healthy, weanling piglets	Basal feed + 1.5 g/kg LHE Basal feed + 1.5 g/kg LDE Basal feed + 1.5 g/kg LHE & LDE	Animals offered LHE diet had lower (P < 0.05) numbers of colonic *Bifidobacterium* and lower populations of cecal and colonic (P < 0.05, P < 0.001, respectively) lactobacilli compared to control diet Supplementation with LDE resulted in lower populations of cecal and colonic (P < 0.05) *Enterobacterium*, cecal (P < 0.05) *Bifidobacterium* and cecal and colonic (P < 0.05, P < 0.001, respectively) *Lactobacillus* compared to control diet Animals offered combination diet had lower (P < 0.05) populations of colonic and cecal *Enterobacterium* and *Lactobacillus* (P < 0.01) compared to control diet	No effects on animal performance Marginal differences in systemic immune response reported in animals fed combination diet	[76]
*L. digitata* extract containing laminarin (0.11 g/kg), and fucoidan (0.89 g/kg)	Healthy, weanling piglets fed a nutritionally-challenged diet	Diet 1: 150g lactose (L)/kg Diet 2: 150 g/kg lactose + 2.8 g/kg seaweed extract (SE) Diet 3: 250g lactose/kg Diet 4: 250g lactose/kg + 2.8 g/kg SE	The inclusion of SE decreased (P < 0.05) fecal *E. coli* counts compared to non-SWE diets Dietary inclusion of SE increased (P < 0.001) *Lactobacillus* counts in pigs fed high L diets	Animals offered seaweed diets had higher (P < 0.01) average daily gain and gain to feed ratio (P < 0.05) Fecal score not affected by dietary inclusion of SE	[81]

## Abbreviations

GIT: Gastrointestinal tract
SCFA: short-chain fatty acids
NAOS: Neoagaro-oligosaccharides
FOS: Fructo-oligosaccharides
FISH: Fluorescence in situ hybridization
PCR: Polymerase chain reaction
DGGE: Denaturing gradient gel electrophoresis
qPCR: Quantitative PCR
